# An Assessment of the Economic Feasibility of Selected Surgeries in the Obstetrics and Gynaecology Department under Community-Based Health Insurance (CBHI) in a Tertiary Care Hospital in South India

**DOI:** 10.1155/2021/1158533

**Published:** 2021-09-25

**Authors:** Jatoveda Haldar, Rajesh Kamath, Kramer Stallone D'lima, Jossil Nazareth

**Affiliations:** ^1^Prasanna School of Public Health (PSPH), Manipal Academy of Higher Education, Manipal, Karnataka, India; ^2^Department of Health Innovation, Prasanna School of Public Health (PSPH), Manipal Academy of Higher Education, Manipal, Karnataka, India; ^3^Finance Department, Kasturba Hospital, Manipal, Karnataka, India

## Abstract

Community-Based Health Insurance (CBHI) is a form of micro health insurance targeted at low-income groups that permits for grouping of assets to tackle the expenses of future, uncertain, health-related circumstances. According to the International Labour Organisation, more than 80% of India's employed nonagricultural population is in the informal sector, implying that they are possibly excluded from receiving health insurance benefits. This is where CBHI comes into play, wherein groups of people belonging to a community define the demand and benefits and pool their resources to provide financial protection to all their members. This study aims to scrutinize the package prices sanctioned by these schemes and compare them with the cost incurred by the hospital. The expense pattern of three surgeries in the Department of Obstetrics and Gynaecology was analysed under three insurance schemes: Arogya Bhagya Yojana, Arogya Karnataka, and Employees' State Insurance Scheme. *Methodology*. A retrospective study was conducted in a 2,032-bedded tertiary care hospital in South India. Patients of abdominal hysterectomy, vaginal hysterectomy, and caesarean section surgeries covered by any of the insurance schemes mentioned above were a part of the inclusion criteria. The patient records were examined from the hospital's Medical Records Department (MRD). The patients' bills were assembled from the inpatient billing department to scrutinize all the expenses associated with each surgery. The variable costs include consumables, medicine, electricity and AC, diagnostics, blood bank materials, doctor's fee, package differences, and others. In contrast, fixed costs include bed cost, equipment cost (purchase + annual maintenance cost), manpower cost-OT, manpower cost-nursing, and allocated indirect costs associated with the medical treatment. These were computed and compared with the package price of respective insurance schemes to determine if the schemes are profit-yielding schemes or loss-yielding schemes, using the data from the finance department. *Results and Conclusion*. It has been observed that the operating loss of the hospital for abdominal hysterectomy, vaginal hysterectomy, and caesarean section under CBHI schemes ranges between 7% and 36%. The highest loss was observed in Arogya Karnataka Scheme for caesarean section surgery (BPL patients). The amount received through these schemes is insufficient to cover the costs acquired by the hospital, let alone make a profit. However, under Arogya Bhagya and ESI Schemes, the hospital has made a profit in covering the variable costs for these surgeries. The study concludes that the hospital is running under loss due to the three Community-Based Health Insurance (CBHI) schemes.

## 1. Introduction

India has witnessed massive headway in the sector of science, technology, and health, notwithstanding financial limitations. According to the RBI, 25.7% of the rural population and 13.7% of the urban population live below the poverty line [1]. Half of the world's poor live in just five countries, with the highest being in India, living beneath the international poverty level in 2015, with 23.88% of its inhabitants below the $1.90-a-day poverty parameter [2–6].

When it comes to health, the Indian Government's expenditure as a percentage of GDP accounts for only 1.15% of GDP for healthcare expansion and evolution [7, 8]. One of the objectives of the National Health Policy 2017 is to increase health expenditure by the government as a percentage of GDP to 2.5% by 2025 [7]. When compared to the BRICS countries, India's expenditure on healthcare is very poor, indicating that most Indians require spending out of their pocket to cover healthcare expenditure [9]. Poorer countries also rely heavily on direct payments, pushing low-income households into poverty [10]. Over 63 million people in India are faced with poverty every year due to healthcare costs [11].

One of the techniques for making healthcare affordable for all is with the help of health insurance [12]. The principal purpose of insurance is to safeguard an individual from economic turmoil because of unforeseen sickness or injury that needs colossal spending [13]. In India, barely 35% of the overall population was safeguarded by health insurance at the end of 2018 [14]. India has been encouraging Community-Based Health Insurance (CBHI) schemes as a segment of the National Rural Health Mission (NRHM) to lessen the load of out-of-pocket spending by the households [15].

The Karnataka state stands 8^th^ in terms of population in the country, with 6.83 crore people (4.91% of the total Indian population) as per 2011 census predictions for 2021. As per Karnataka Integrated Public Health Policy 2017, health spending has increased in the last 15 years. However, the proportion of health expenditure to the GSDP has decreased from 1.46 in 2000-2001 to 1.0 in 2013-2014, while the percentage of total state expenditure spent on health has remained constant [16].

In 2017, Karnataka launched the Arogya Bhagya Yojana scheme to impart healthcare at liberty to its citizens. In this scheme, the beneficiaries can avail medical and surgical treatments from both private and government hospitals. The Arogya Bhagya scheme looks at delivering healthcare covering 14 million families in the state. Thus, about 1.4 crore households will acquire cashless treatment via this scheme, protecting five household members on average [17]. This study focused on the characteristics, profits, and requisition method of the Arogya Bhagya scheme in depth.

Karnataka is the first state in the country to announce and apply Universal Health Coverage to protect the people of Karnataka from poverty. The Arogya Karnataka scheme includes primary, secondary, and tertiary healthcare. The health schemes such as Vajpayee Arogyashree, Yeshaswini Scheme, Rajiv Arogya Bhagya Scheme, Rashtriya Swasthya Bima Yojana (RSBY), including RSBY for senior citizens, Rashtriya Bal Swasthya Karyakram (RBSK), Mukhyamantri Santwana Harish Scheme, Indira Suraksha Yojana, and Cochlear Implant Scheme have coincided under Arogya Karnataka scheme [18]. This scheme was rolled out in Karnataka in parts; the first part included ten vital hospitals. UHC will be advantageous for patients, but the question that will still be unanswered is whether the medical expenses borne by the hospitals would be compensated or not.

The ESI Scheme was first inaugurated in Kanpur by the Prime Minister Pandit Jawaharlal Nehru on 24th February 1952. It is designed to protect employees as defined in Employees' State Insurance Act, 1948, against illnesses, maternity, disablement, and death due to employment injury and provide medical care to the insured persons and their families. The ESI Corporation decided to set up a subregional office at Mangalore to mitigate the hurdles of employers and the insured persons. This office exercises jurisdiction over 12 centers spread in Dakshina Kannada and Udupi districts. All over 2000 factories/establishments have been included under ESI Scheme, and about 2,20,000 insured individuals are receiving benefits under the scheme [19].

Abdominal hysterectomy, vaginal hysterectomy, and caesarean section surgeries were chosen for the study as there has been an increase in caesarean section deliveries in private hospitals from 40.3% to 52.5%, according to the latest National Family Health Survey (NFHS-5) data for Karnataka. A study by Prusty, Choithani, and Gupta revealed that Andhra Pradesh (6%), Telangana (5.5%), and Karnataka (3%) had a comparatively higher prevalence of hysterectomy than other states. This study underlines the hurdles faced by the hospital under CBHI, specifically for these three surgeries under the Arogya Bhagya Yojana, Arogya Karnataka, and Employees' State Insurance schemes.

## 2. Objectives

The objectives of the present study were to perform a cost analysis of selected surgeries and to compare the expense patterns acquired by the hospital with the package rates, thus, to assess the economic feasibility of the schemes covering selected surgeries in the Obstetrics and Gynaecology Department of a tertiary care hospital in South India.

## 3. Methods and Materials

The study was conducted following the approval by the Institutional Ethics Committee (IEC) on 30th January 2021 (IEC: 962/2020). A bottom-up approach using activity-based costing (ABC) was taken up to approximate the expense of selected obstetrics and gynaecology surgeries in a large, 2,032-bedded tertiary care teaching hospital.

The study was executed in two steps. The first step focused on estimating the costs of the services and materials included in each procedure's hospital package. The primary obstacle was in scrutinizing the patients' bills to figure out the expense of services and materials that the patients opted for during their stay at the hospital but were not included in the hospital package. For this, the patients' bills were gathered for the three CBHI schemes managed at the hospital.

The data were gathered for three months, starting from February 2021 to April 2021; all the surgeries were held between April 2019 and March 2020 under the three insurance schemes selected for analysis.

This analysis started by scrutinizing the hospital package to pick various expense heads. It included consumables, medicine, electricity and AC, diagnostics, blood bank materials, doctor's fee, bed costs, equipment costs (purchase + annual maintenance cost), manpower cost-OT, manpower cost-nursing, and allocated indirect costs.

Later, the bills of each surgery were scrutinized to find out different expense heads not included in the hospital package of which the patients were charged.

The manpower cost is deliberated as unit cost per minute for the assistance/services of staff members, such as surgeons, anaesthesiologists, nurses, paramedical staff, and support staff. The finance department of the hospital imparted accounting details of consumable costs per surgery in depth.

The cost incurred by the hospital for the surgeries done was divided into the following subtypes:

- Bed cost: it includes the cost of the bed, electricity-fan, maintenance of ward (housekeeping), diet, linen cost, and water cost
- Consumables
- Electricity and AC
- Equipment cost (purchase + annual maintenance)
- Medicine (sat pharmacy + anesthesia and OT drugs)
- Diagnostics
- Blood bank materials
- Doctor's fee
- Manpower cost-OT
- Manpower cost-nursing

Indirect costs for all categories mentioned above per procedure were summed to obtain the total cost per treated patient. Finally, in the second step, the total cost was compared with the amount approved by each insurance scheme.

## 4. Result and Analysis

The population under study was patients of abdominal hysterectomy, vaginal hysterectomy, and caesarean section surgeries in Obstetrics and Gynaecology Department under Community-Based Health Insurance, particularly Arogya Bhagya, Arogya Karnataka, and ESI in a tertiary care hospital in South India.

**Figure 1:**
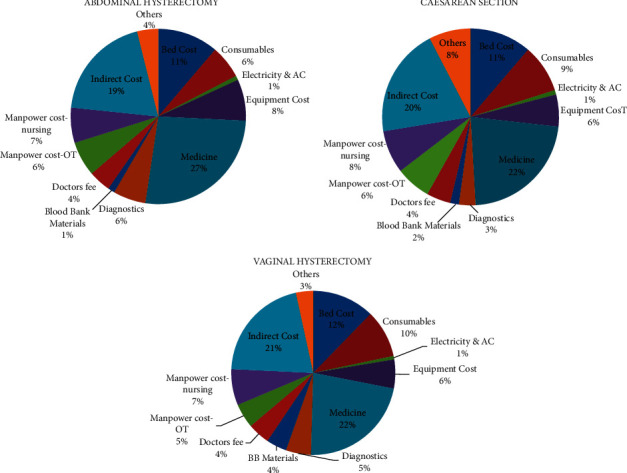
Overall cost composition of OB/GYN surgeries under hospital package. Cost distribution for (a) abdominal hysterectomy, (b) caesarean section, and (c) vaginal hysterectomy.

Table 1 and Figure 1 indicate the overall cost structure of the hospital package of obstetrics and gynaecology surgeries, showing that most of the costs come under medicine, indirect cost, and bed cost.

From Figure 1, it has been observed that, under abdominal hysterectomy, caesarean section, and vaginal hysterectomy, the contributing factor for high expenses is medicine cost and indirect cost (12.7% of hospital bill amount).

Table 2 indicates the total expense structure of OB/GYN surgeries under different schemes.

**Figure 2:**
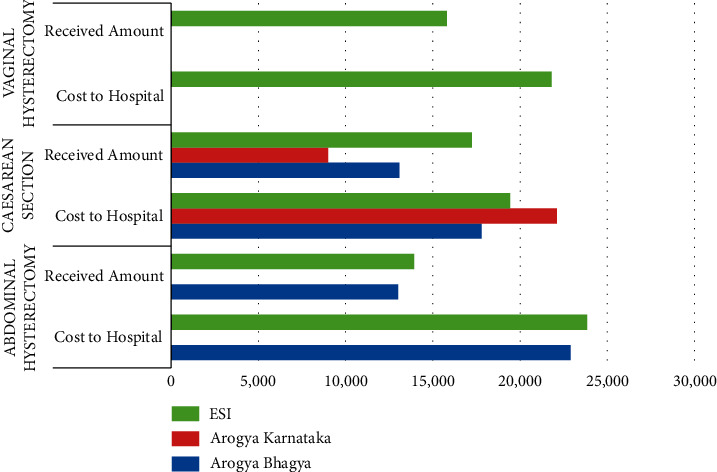
Loss from abdominal hysterectomy, caesarean section, and vaginal hysterectomy under each scheme.

Table 3 and Figure 2 show the total cost incurred to the hospital and the amount received for each surgery under all the schemes included in the study.

For all schemes under abdominal hysterectomy, the amount received was lesser than the total cost incurred to the hospital. The loss margins under Arogya Bhagya and ESI were almost equal, with 43.17% and 41.56%, respectively (Table 3 and Figure 2). It was observed that the patients treated under the Arogya Bhagya scheme are of higher complexity which increases the length of stay and cost of the medicine compared to the ESI scheme. It incurred a loss to the hospital as the amount received would be based on the rates under CGHS. There were no patients for abdominal hysterectomy under Arogya Karnataka (BPL) as per the inclusion criteria for this study.

Under caesarean section, the received amount was lesser than the total cost incurred to the hospital. The lowest loss margin was under the ESI scheme with 11.26%, and the highest loss margin was under the Arogya Karnataka scheme with 59.29% (Table 3 and Figure 2). It was observed that the patients treated under the Arogya Karnataka scheme have the privilege of getting the treatment and other facilities without any out-of-pocket expenditure compared to other schemes. This is due to the fact that the Arogya Karnataka scheme provides medical facilities to people below the poverty line, and the patients covered under this scheme are mainly from rural areas [20].

Under vaginal hysterectomy, the received amount was lesser than the total cost incurred by the hospital. The loss margin under the ESI scheme was 27.53% (Table 3 and Figure 2). It is a complex surgery requiring more time in the OT, thus increasing the indirect manpower cost and medicine cost. There were no patients for vaginal hysterectomy under Arogya Bhagya and Arogya Karnataka (BPL) as per the inclusion criteria for this study.

In Table 4, we can see a contribution profit ranging from 6% to 26% when it comes to variable costs. In comparison, fixed costs result in a loss under the schemes, ranging from 7% to 36%. It is found that bed cost, equipment cost (purchase + annual maintenance cost), manpower cost-OT, manpower cost-nursing, and allocated indirect costs incur more costs to the hospital, which result in such losses. Thus, efforts should be taken to control these areas in particular. Also, in the Arogya Karnataka scheme, more loss is incurred to treat below poverty line patients. Neither variable nor fixed costs contribute towards any profit to the hospital under this scheme.

## 5. Discussion

India has the largest private healthcare system, with nearly 87% of the healthcare services provided by the private sector and the out-of-pocket expenditure accounting for 70% of the GDP spent on healthcare [21–24]. Despite this, the private sector has faced many challenges due to the sector's poor financial health. This is likely to be impacted further due to the launch of certain insurance schemes by the government, as the reimbursement tariffs cover not more than 40–80% of the total cost incurred to the hospital. It has been expected that there will be a 15–25% decline in the ARPOBs (average revenue per occupied bed day) and a 25–50% decline in EBITDA in multispecialty NABH (National Accreditation Board for Hospitals & Healthcare Providers) accredited hospitals in case no change is made in the current operating model [25]. It is hence imperative to address the concerns of financial sustainability of the private sector, as well as affordability for the patient.

In a study conducted by the Health Services Committee of the Federation of Indian Chambers of Commerce and Industry (FICCI), it has been estimated that the ROCE (return on capital employed) would drop by 60% if the current operating model continues. A collaborative study by IIM Bangalore, the Karnataka government, and the National Accreditation Board for Hospitals and Healthcare Providers (NABH) has established that the reimbursement provided under government health insurance schemes is not sufficient when compared to the actual cost of medical procedures. In 2018, private hospitals in Kerala threatened to end government schemes as not only are the rates offered by the government less but the reimbursements are often received after four to five months [26].

These findings are in agreement with the present study, wherein the hospital is making a loss ranging from 7% to 36% under various Community-Based Health Insurance (CBHI) schemes. The government has “frozen” the rates of procedures and surgeries for these insurances and is unlikely to change them despite protests from the private health sector. The government's reimbursements to the hospitals under the vast new health insurance schemes are insufficient, wherein even during the COVID-19 pandemic, the insurance amounts were capped [27]. As there are not many studies covering the Department of Obstetrics and Gynaecology under CBHI schemes, the findings of this study may be used for further research or for building better insurance models.

The Ayushman Bharat-National Health Protection Scheme (AB-NHPS), launched in 2018, is one of the forerunner programs in the health sector. The rates prescribed by the government schemes are “base rates,” which can be increased by even 30% for some exceptions. Despite this fact, hospitals have been running at a loss by taking part in the Central Government Health Scheme (CGHS) and other insurance schemes but have been managing by using their corporate social responsibility (CSR) funds. This will not be possible when ten crore families become competent for these low rates [28].

As these CBHI schemes have been around for quite some time, people have become more aware of these schemes, and as a result, there is an increase in the utilization of health services, particularly in the private sector [29–31].

Hence, the clinical implications of this study include using substandard material for patients under these schemes to reduce cost and possible public pressure in the future towards reducing cash tariffs of private healthcare providers as the general public has now become increasingly aware of the price differences. This study will also help policymakers understand the plight of private hospitals, which may help them bring about new modifications in the existing operational model.

## 6. Conclusion

This study involved the analysis of the cost incurred to the hospital and the package amount received from Community-Based Health Insurance schemes for patients treated in a tertiary care teaching hospital. The analyses were carried out on 90 cases of obstetrics and gynaecology surgeries selected across three Community-Based Health Insurance schemes. The cost was assigned per-patient basis. Costs were categorized into different categories: consumables, medicines, manpower, electricity, equipment costs, etc. Then, the total costs were compared to the amount received to ascertain whether the hospital is making any loss or profit. A significant number of cases had a high cost of medicines followed by indirect cost, bed cost, and equipment cost. For all the surgeries approved, the amount received from the insurance schemes was lesser than the cost incurred to the hospital. Adding to the burden, lengthened stay of the patient at the hospital because of posttreatment complexity is not included in the treatment package by insurance schemes. The operating loss was in the range of 7%–36%. This study draws the inference that Community-Based Health Insurance (CBHI) schemes are financially infeasible for the Obstetrics and Gynaecology Department of the hospital.

## Figures and Tables

**Table 1:** Total package cost of each surgery.

	Abdominal hysterectomy	Caesarean section	Vaginal hysterectomy
Bed cost	2,675	2,229	2,675
Consumables	1,461	1,747	2,056
Electricity and AC	188	163	150
Equipment cost	1,821	1,156	1,244
Medicine	6,341	4,415	4,874
Diagnostics	1,437	604	1,089
Blood bank materials	298	314	876
Doctor's fee	971	872	951
Manpower cost-OT	1,529	1,274	1,052
Manpower cost-nursing	1,549	1,549	1,549
Indirect cost	4,641	3,949	4,549
Others	928	1,508	743
Total (₹)	**23,839**	**19,780**	**21,808**

**Table 2:** Total cost to the hospital under abdominal hysterectomy, caesarean section, and vaginal hysterectomy.

	Abdominal hysterectomy		Caesarean section			Vaginal hysterectomy
	Arogya Bhagya	ESI	Arogya Bhagya	Arogya Karnataka	ESI	ESI
Bed cost	**2,675**	**2,675**	**2,229**	**2,229**	**2,229**	**2,675**
Consumables	814	1,461	2,014	1,665	1,562	2,056
Electricity and AC	188	188	163	163	163	150
Equipment cost	1,821	1,821	1,156	1,156	1,156	1,244
Medicine	7,355	6,341	3,459	4,982	4,804	4,874
Diagnostics	1,359	1,437	144	1,364	303	1,089
Blood bank materials	—	298	—	943	—	876
Doctor's fee	933	971	816	944	855	951
Manpower cost-OT	1,529	1,529	1,274	1,274	1,274	1,052
Manpower cost-nursing	1,549	1,549	1,549	1,549	1,549	1,549
Indirect cost	4,361	4,641	3,368	4,623	3,857	4,549
Others	320	928	1,630	1,217	1,677	743
Total (₹)	**22,904**	**23,839**	**17,802**	**22,109**	**19,429**	**21,808**

**Table 3:** Loss under abdominal hysterectomy, caesarean section, and vaginal hysterectomy.

Scheme	Abdominal hysterectomy			Caesarean section			Vaginal hysterectomy		
	Cost to hospital (₹)	Received amount (₹)	Loss (%)	Cost to hospital (₹)	Received amount (₹)	Loss (%)	Cost to hospital (₹)	Received amount (₹)	Loss (%)
Arogya Bhagya	**22,904**	**13,016**	**43.17**	**17,802**	**13,088**	**26.48**	**—**	**—**	**—**
Arogya Karnataka	**—**	—	—	**22,109**	9,000	59.29	**—**	—	—
ESI	**23,839**	13,931	41.56	**19,429**	17,241	11.26	**21,808**	15,804	27.53

**Table 4:** Costing for calculation of contribution, total variable, and fixed cost and profit or loss%.

		Abdominal hysterectomy		Caesarean section			Vaginal hysterectomy
Sl. no.	Particulars	Arogya Bhagya	ESI	Arogya Bhagya	Arogya Karnataka	ESI	ESI
*A*	Hospital price	34,338	38,752	26,517	36,399	30,370	35,817
	Less: variable costs						
1	Consumables	814	1,676	2,014	1,665	1,562	2,056
2	Medicine	7,355	6,002	3,459	4,982	4,804	4,874
3	Electricity and AC	188	188	163	163	163	150
4	Diagnostics	1,359	1,463	144	1,364	303	1,089
5	BB materials	0	298	0	943	—	876
6	Doctor's fee	933	971	816	944	855	951
7	Package difference	21,322	24,821	13,429	27,399	13,129	20,012
8	Other variable costs (₹)	320	1,130	1,630	1,217	1,677	743
*B*	**Total variable cost** (₹)	32,291	36,549	21,655	38,677	22,493	30,751
*C*	**Contribution margin ( ***A*−*B ***)**	**2,047**	**2,203**	**4,862**	**−2,278**	**7,877**	**5,066**
	Contribution margin % (*C*/*A*)	6%	6%	18%	−6%	26%	14%
	Less: fixed costs						
1	Bed cost	2,675	2,675	2,229	2,229	2,229	2,675
2	Equipment cost	1,821	1,821	1,156	1,156	1,156	1,244
3	Manpower cost-OT	1,529	1,529	1,274	1,274	1,274	1,052
4	Manpower cost-nursing	1,549	1,549	1,549	1,549	1,549	1,549
5	Allocated indirect costs	4,361	4,922	3,368	4,623	3,857	4,549
*D*	**Total fixed costs** (₹)	**11,935**	**12,496**	**9,576**	**10,831**	**10,065**	**11,069**
*E*	**Operating profit/loss ( ***C*−*D ***)**	**−9,888**	**−10,293**	**−4,714**	**−13,109**	**−2,188**	**−6,003**
	Operating profit/loss % (*E*/*A*)	−29%	−27%	−18%	−36%	−7%	−17%

## Data Availability

The data are available from the authors on request.
